# A Knockout of the IFITM3 Gene Increases the Sensitivity of WI-38 VA13 Cells to the Influenza A Virus

**DOI:** 10.3390/ijms25010625

**Published:** 2024-01-03

**Authors:** Natalya Eshchenko, Mariia Sergeeva, Evgenii Zhuravlev, Kira Kudria, Elena Goncharova, Andrey Komissarov, Grigory Stepanov

**Affiliations:** 1 Institute of Chemical Biology and Fundamental Medicine, Siberian Branch of the Russian Academy of Sciences, Novosibirsk 630090, Russia; eschenko96@gmail.com (N.E.); mari.v.sergeeva@gmail.com (M.S.); goncharova-ep@rambler.ru (E.G.); a.b.komissarov@gmail.com (A.K.); stepanovga@niboch.nsc.ru (G.S.); 2 Smorodintsev Research Institute of Influenza, Ministry of Health of the Russian Federation, St. Petersburg 197376, Russia; kira336@yandex.ru

**Keywords:** IFITM, influenza A virus, human cells, WI-38, genome editing, CRISPR/Cas9, RNA-seq

## Abstract

One of the ways to regulate the sensitivity of human cells to the influenza virus is to knock out genes of the innate immune response. Promising targets for the knockout are genes of the interferon-inducible transmembrane protein (IFITM) family, in particular the *IFITM3* gene, whose product limits the entry of a virus into the cell by blocking the fusion of the viral and endosomal membranes. In this study, by means of genome-editing system CRISPR/Cas9, monoclonal cell lines with an *IFITM3* knockout were obtained based on WI-38 VA13 cells (human origin). It was found that such cell lines are more sensitive to infection by influenza A viruses of various subtypes. Nevertheless, this feature is not accompanied by an increased titer of newly formed viral particles in a culture medium.

## 1. Introduction

Influenza viruses are the principal human respiratory pathogens that cause seasonal epidemics as well as unpredictable outbreaks and pandemics [1]. Seasonal influenza occurs globally and is estimated to affect one in five unvaccinated children and one in ten unvaccinated adults [2]. At present, vaccination remains the most effective method for counteracting seasonal infections and is essential to ensure preparedness for annual influenza epidemics [3,4,5]. In the manufacturing process of the vast majority of flu vaccines, a traditional technology is used—cultivation of the virus in chicken embryos—which was developed more than half a century ago [6,7]. Although the technology involving chicken embryos is very reliable and safe, there are still some unsolvable problems associated with this production system. In particular, an occasional antigenic mismatch between egg-adapted viruses and circulating viruses as well as allergic reactions to egg whites when a ready-made vaccine is administered to a patient can occur [8,9,10]. The speed of technological process is also crucial. The full production cycle of a vaccine involving chicken embryos is 6 months; this state of affairs substantially reduces manufacture flexibility and makes the task of a rapid response to an epidemiological threat impossible [11].

In the last two decades, research into vaccine manufacturing approaches based on cell cultures has been gaining popularity among scientific laboratories and biotech companies [12,13]. Such production systems partially eliminate the above disadvantages and offer several potential advantages, including the ability to efficiently produce sufficient amounts of a wider range of strains, rapid scalability and high antigenic specificity of a vaccine virus to seasonal strains [14,15]. In addition, the more isolated process for the production of cell-based vaccines and the possibility of detailed testing of cell banks for contamination by random agents considerably reduces the risk of contamination in finished vaccines [16,17].

The main criterion for choosing a cell line for the production of inactivated influenza vaccines is the sensitivity and ability of the cells to support propagation of a wide variety of influenza viruses at high titers. To date, the mammalian Madin–Darby canine kidney (MDCK) cell line and the African green monkey kidney (Vero) cell line have found the largest number of applications in this field [18,19,20]. Nonetheless, a virus produced in cell lines of animal origin can accumulate adaptive mutations and may have an altered glycosylation profile. These factors negatively affect immunological properties of the resulting vaccines [21,22].

The technology for producing vaccine strains of an influenza virus in cell lines of human origin makes sense and is promising [23]. On the other hand, practical use of such cell lines is limited because they generate only low titers of vaccine strains. The limiting factor is the system of innate immune responses functioning within human cells [24,25]. One possible way to eliminate this phenomenon is a targeted knockout of genes encoding components of the innate immune response [26]. Therefore, in the present work, by means of genome-editing system CRISPR/Cas9, monoclonal cell lines with an *IFITM3* knockout were created from cells of human origin.

## 2. Results

### 2.1. The Choice of a Target Gene and Cell Line for the CRISPR/Cas9 Editing

The *IFITM3* gene, which is a member of the family of interferon-induced transmembrane proteins (IFITMs) [27], was chosen as the target gene for the knockout. IFITM3, along with proteins IFITM1 and IFITM2, has an antiviral activity [28]. IFITM3 inhibits the fusion of viral and cellular membranes, thereby limiting the penetration of the virus into the cytoplasm and virus replication [29,30]. It has been shown that IFITM3 depletion profoundly decreases the antiviral processes in virus-infected cells and overexpression of IFITM3 increases resistance to infection with influenza A viruses [31].

**Figure 1:**
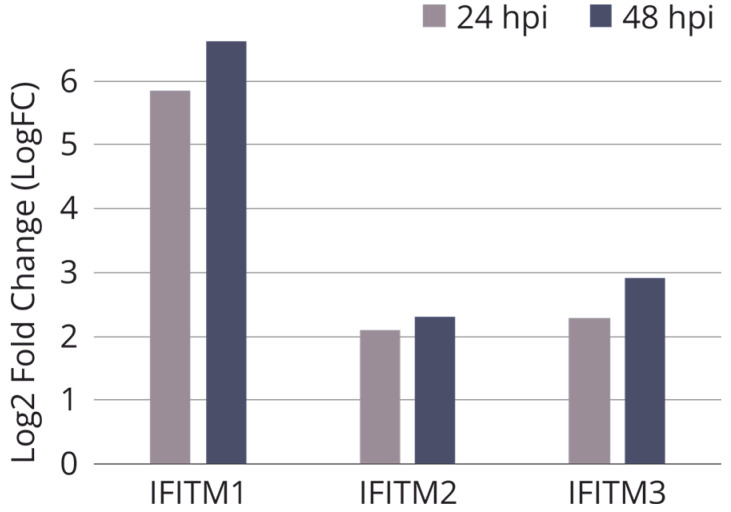
Relative expression of *IFITM* genes in influenza A infected WI-38 VA-13 cells after 24 h (24 hpi) and 48 h (48 hpi) according to results of our transcriptomic analysis. Log2 fold change (LogFC) values were generated for transcriptome sequencing samples by comparing the average RPKM values of genes at each timepoint of infection vs. uninfected cells.

To knock out *IFITM3*, immortalized human embryonic lung fibroblast line WI-38 VA13 was chosen (subline 2RA from the Collection of Cultures of Vertebrate Cells at the Institute of Cytology, the Russian Academy of Sciences). This cell line was derived from WI-38 cells, which have been employed to design vaccines against several viral diseases [32]. Cell subline WI-38 VA13 is characterized by a known transformation history, which allows for its use as a substrate for vaccine production. According to results of our transcriptomic analysis, at 24 and 48 h after infection of WI-38 VA13 cells with influenza virus A/PR/8/34 (H1N1), the expression level of the *IFITM1*, *2* and *3* genes increases relative to uninfected cells (Figure 1).

### 2.2. Construction of Cell Lines with Knocked Out IFITM3

**Figure 2:**
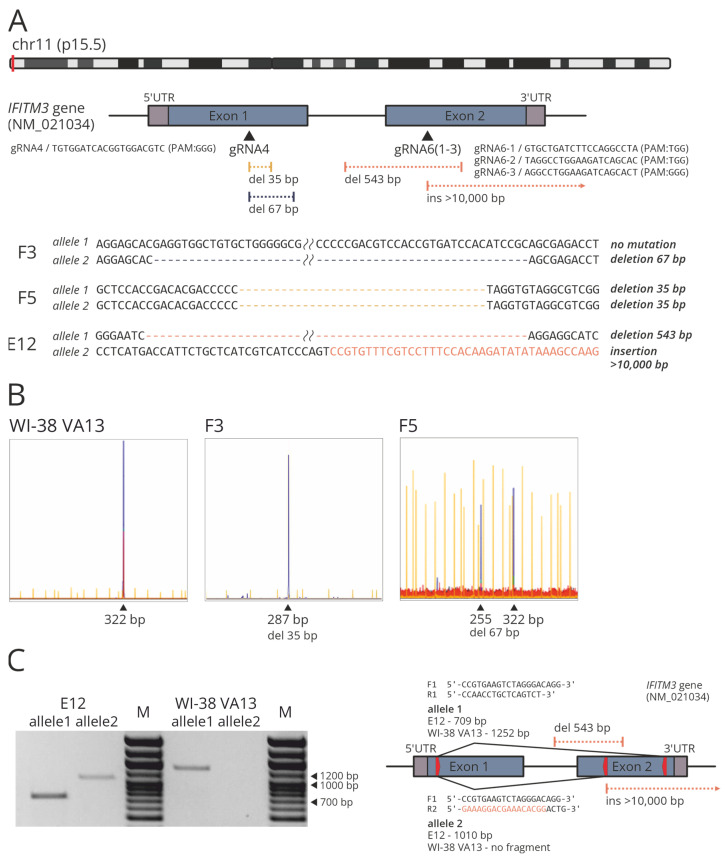
(**A**) Scheme of the location of protospacers and identified CRISPR/Cas9-mediated mutations in target region of the *IFITM3* gene. (**B**) IDAA analysis of modified (F3, F5) and control WI-38 VA13 cells. Amplicon and indel sizes determined (in bp) are shown above the most prominent peaks. (**C**) PCR products obtained from the gDNA extracted from modified (E12) and control WI-38 VA13 cells, with the primers flanking the extended deletion in clone E12 (F1_R1/allele 1) and with the reverse primer that was partly complementary to the *IFITM3* gene and partly to the plasmid PX458 region integrated into the genome (F1_R2/allele 2). M, DNA molecular weight marker. The scheme shows the location of the primers (red arrows) and the expected length of the products for modified (E12) and control WI-38 VA13 cells.

The procedure for obtaining monoclonal cell lines by means of the CRISPR/Cas9 system included standard stages: transfection of WI-38 VA13 cells with vectors constructed based on plasmid pX458 (Addgene, cat. # 48138), sorting of GFP-positive cells, subcloning of a cell population, selection of monoclonal cell lines with mutations in a target region of *IFITM3*, determination of the mutations in each allele by TA-cloning of PCR products and Sanger sequencing and a comparison of proliferation rates and selection of monoclonal cell lines using xCELLigence (ACEA Biosciences) technology [33]. Thus, three monoclonal lines of viable actively dividing cells were obtained with mutations in the target region of the *IFITM3* gene (Figure 2).

Clone F5 was found to carry two mutant alleles with 35-nucleotide (nt) deletions which may indicate a frame shift in both copies of the target gene (Figure 2A). Clone F3 carries only one allele with the mutation (namely, a 67-nt deletion) and the second allele corresponds to the wild type. Clone E12 proved to be a carrier of a 543-nt deletion and of an insertion of a >10 kbp part of the PX458 plasmid. Mutations in clones F3 and F5 were confirmed via indel detection by amplicon analysis (IDAA) (Figure 2B) [34]. The presence of an extended deletion in clone E12 was corroborated by PCR analysis of genomic DNA with flanking primers (Figure 2C). To confirm the insertion, a reverse primer was utilized that was partly complementary to the *IFITM3* gene and partly to the plasmid PX458 region integrated into the genome (Figure 2C).

**Figure 3:**
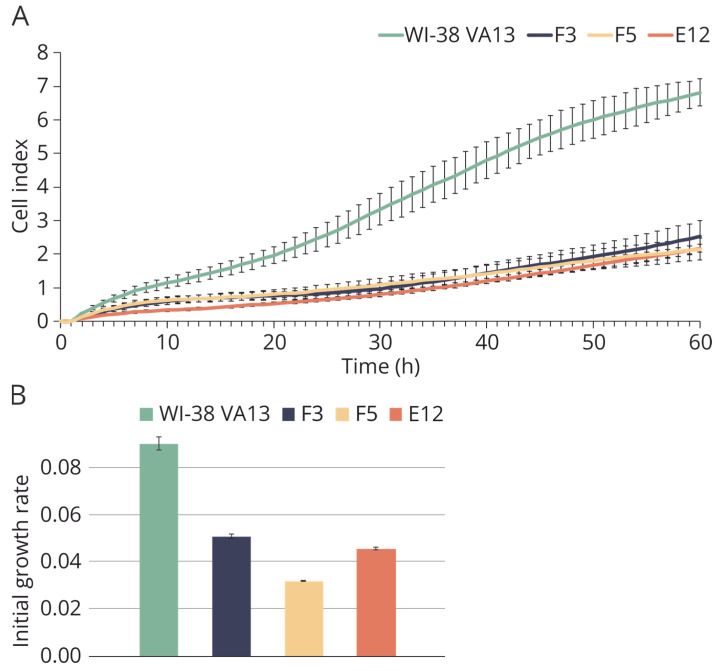
Growth characteristics of modified and control WI-38 VA13 cells. (**A**) Growth curves of cells defective in *IFITM3* gene (F3, F5, E12) and control WI-38 VA13 cells according to xCELLigence data. (**B**) Values of initial growth rate of cell lines during first 48 h.

Using xCELLigence technology, the selected monoclonal cell lines were proven to be viable, but their proliferation rate was lower than that of the control cell line (Figure 3).

### 2.3. Evaluation of IFITM3 mRNA Expression

**Figure 4:**
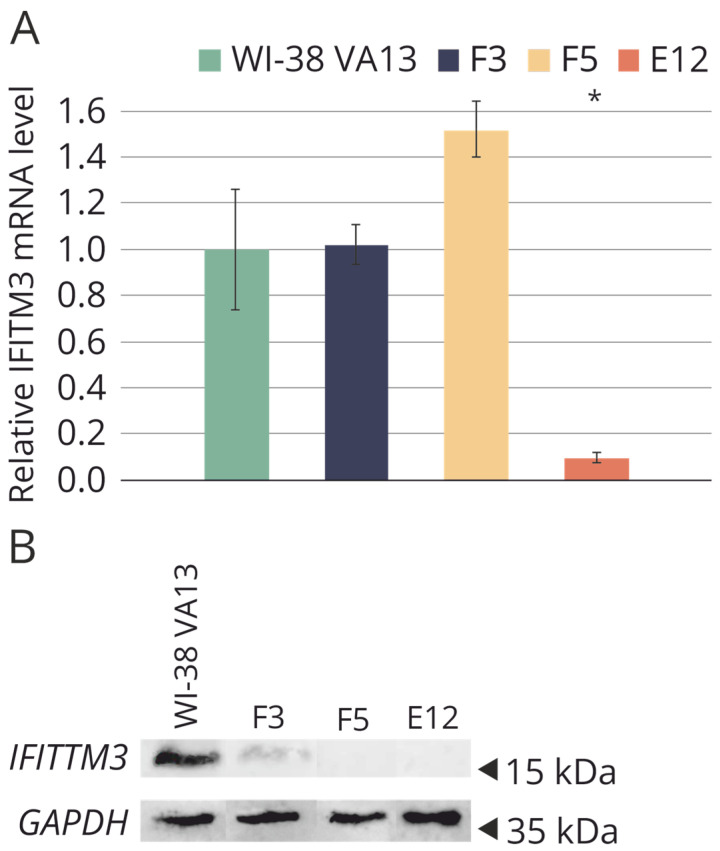
(**A**) Relative basic level of *IFITM3* mRNA in original WI-38 VA13 cells and in the clones with depressed *IFITM3* gene activity (F3, F5, E12). Average value of a change in the expression level relative to reference GAPDH and 18S rRNA is presented and standard deviation was calculated from three independent experiments by data on three independent experiments. * Difference between modified lines and WI-38 VA13 cells is statistically significant at *p* < 0.05 (unpaired student’s *t*-test) (**B**) Western blot analysis of IFITM3 protein level in original WI-38 VA13 cells and monoclonal lines with detected mutations in the *IFITM3* gene.

To assess the level of mRNA expression, real-time reverse transcription (RT) PCR was carried out (Figure 4A). It was demonstrated that the mRNA level of the *IFITM3* gene is significantly reduced in clone E12 compared to the original cell line, likely because of the presence of the extensive mutations in both alleles. The high mRNA level of *IFITM3* in clones F3 and F5 may be explained by the fact that the presence of mutations detected in the clones did not interfere with the synthesis of full-size but incorrect *IFITM3* gene mRNA or its short forms.

Western blot analysis was performed to determine the presence or absence of the target protein in the selected clones (Figure 4B). In the original cell line WI-38 VA13, a high level of IFITM3 expression was confirmed. Mutations in clones F5 and E12 caused the absence of the protein product in the cells. In clone F3, synthesis of the IFITM3 protein was observed, apparently due to the presence of a the nonmutant allele, but the protein level was lower than that in the control cell line.

**Figure 5:**
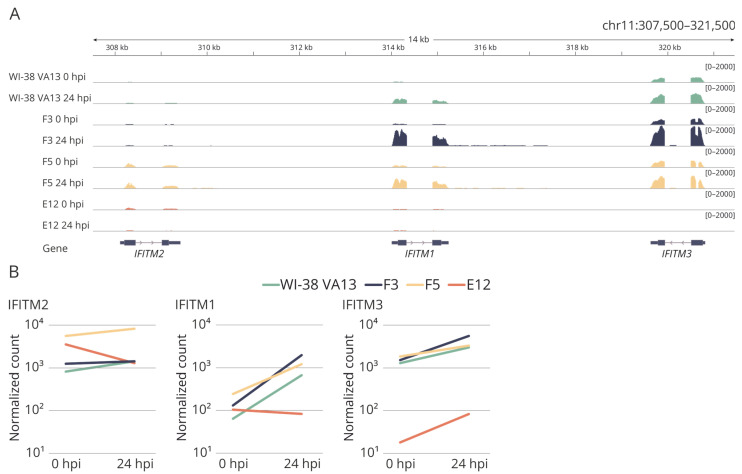
(**A**) The coverage tracks of the genome fragment encoding *IFITM2*, *IFITM1* and *IFITM3* genes for the original WI-38 VA13 cells and clones with mutations (F3, F5, E12) before infection (0 hpi) and at 24 h (24 hpi) after influenza A virus infection. (**B**) Expression of *IFITM2*, *IFITM1*, *IFITM3* genes in original WI-38 VA13 cells and clones with mutations (F3, F5, E12) before infection (0 hpi) and at 24 h (24 hpi) after influenza A virus infection. The normalized counts of mapped reads averaged for two biological repeats are shown.

Mutations in the clones were corroborated by an analysis of transcriptome sequencing data (Figure 5). A decrease in the *IFITM3* mRNA level in clones with mutations was successfully verified. Additionally, mutual regulation of transcriptional activity was demonstrated among genes within the IFITM family. For instance, in clone E12, along with the knockout of *IFITM3*, almost complete suppression of the transcriptional activity of the *IFITM1* gene was observed (Figure 5B).

The magnitude of the observed changes in gene expression at 24 h after influenza A virus infection of the cells is summarized in Table 1. The most significant alterations as compared to parental cells were noted in the E12 cell line.

**Figure 6:**
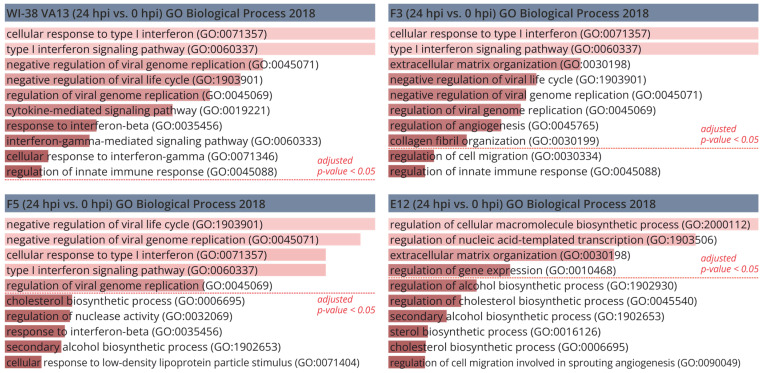
Gene ontology analysis of the most differentially expressed genes in original WI-38 VA13 cells and clones with mutations (F3, F5, E12) at 24 h after influenza A virus infection (relative to uninfected cells). Bar charts showing the top 10 GO terms for biological process, ranked by fold enrichment following analysis of the most differentially expressed genes.

In the cell clones with mutations in *IFITM3*, functional analysis of the genes that are most differentially expressed (relative to uninfected cells) revealed that in cell lines F3 and F5, the enrichment with functional terms was similar to that in the original cell line WI-38 VA13. These functional terms are characteristic of a viral infection. On the other hand, in the set of differentially expressed genes of monoclonal cell line E12 there was enrichment with terms not directly related to the antiviral response: biosynthesis of macromolecules and transcription processes (Figure 6). This result was suggestive of a disturbance in the activation of the innate-immune-response cascade specifically in the clone with impaired expression of the two genes of the IFITM family.

**Figure 7:**
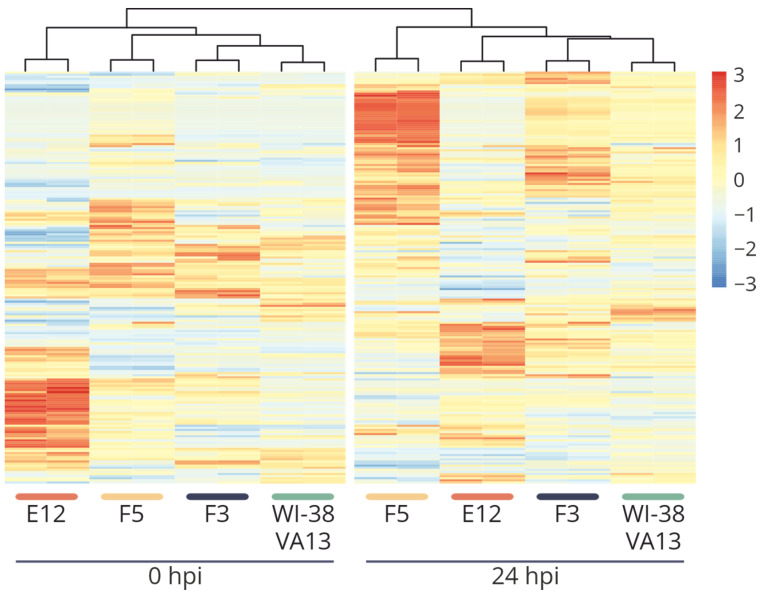
Heat map of scaled normalized expression values of genes of the “response to the virus” group (GO: 0009615) for two biological repeats of original WI-38 VA13 cells and clones with mutations (F3, F5, E12) before infection (0 hpi) and at 24 h (24 hpi) after influenza A virus infection.

Examination of the “response to virus” functional group (GO:0009615) allowed us to confirm this supposition: results of the examination, presented in the form of a heat map, indicated significant differences of cell clones F5 and E12 from the control cell line (Figure 7). At the same time, in monoclonal cell line F5 (a knockout of only *IFITM3*), a tendency toward overexpression of some antiviral-response genes was documented, whereas in cell line E12 (combined suppression of both *IFITM3* and *IFITM1*) we noted reduced activity of the genes of this category.

**Figure 8:**
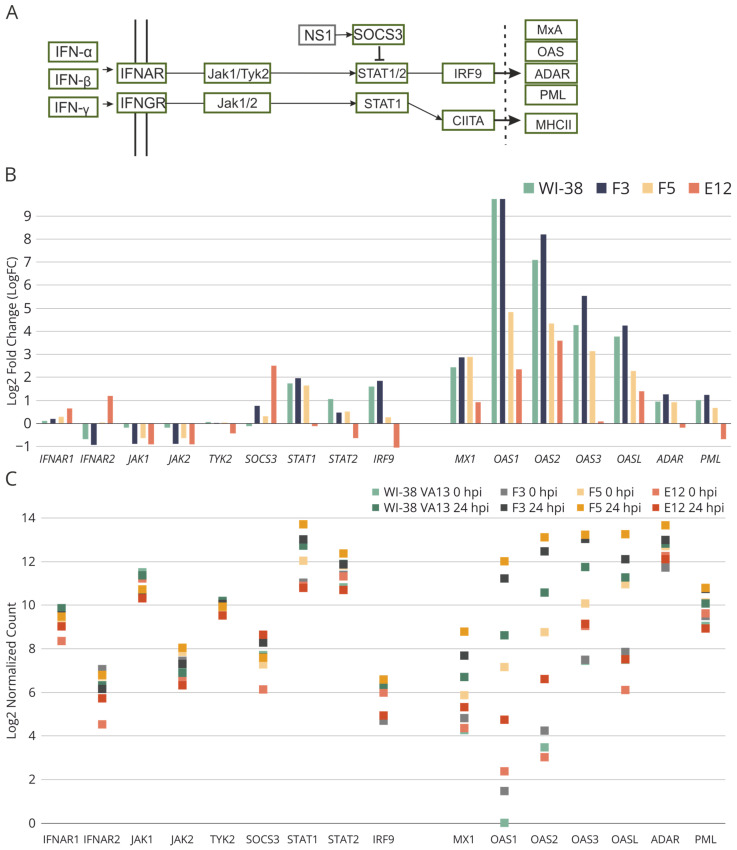
(**A**) Fragment of the scheme of molecular interactions during influenza A virus infection involved in the regulation of Jak-STAT signaling pathway. (**B**) Relative expression of genes involved in the Jak-STAT signaling pathway in influenza A infected WI-38 VA-13 cells at 24 h after influenza A virus infection, according to results of our transcriptomic analysis. Log2 fold change (LogFC) values were generated for transcriptome sequencing samples by comparing the average RPKM values of genes at 24 of infection vs. uninfected cells. (**C**) Expression of genes involved in the Jak-STAT signaling pathway in original WI-38 VA13 cells and clones with mutations (F3, F5, E12) before infection (0 hpi) and at 24 h (24 hpi) after influenza A virus infection. The normalized counts of mapped reads averaged for two biological repeats are shown.

A detailed analysis of the most differentially expressed genes within obtained cell lines identified a gene set directly related to the Jak–STAT signaling pathway: *SOCS3*, *STAT1*, *STAT2* and *IRF9* (Figure 8). In monoclonal cell line E12, the results of transcriptomic analysis revealed disturbances in the activation of a group of genes of the Jak–STAT cascade as compared with control WI-38 VA13 cells (Figure 8B). For several other key genes in the pathways of response to influenza A virus infection, aberrations (from the control) in regulation were also observed in both uninfected monoclonal cell lines and in virus-infected cells (Figure 8C). From these results, we can assume that a knockout of stand-alone genes of the innate immune response leads to dysregulation of the antiviral response. The combined downregulation (of the activity of genes from the IFITM family) seen in the E12 monoclonal cells induces substantial disturbances of the innate-immune-response cascade.

### 2.4. Sensitivity of WI-38 VA13 IFITM3^−/−^ Cell Lines to Influenza Infection

**Figure 9:**
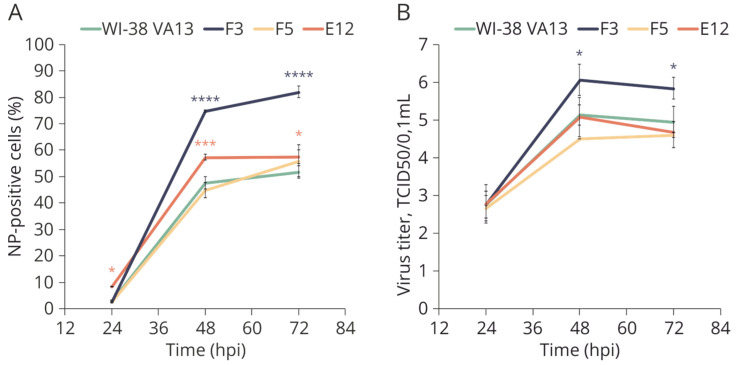
Growth curves of A/PR/8/34 (H1N1) influenza virus in original WI-38 VA13 cells (WI-38 VA13) and clones with mutations (F3, F5, E12). (**A**) Number of productively infected cells expressing influenza virus nucleoprotein, measured by flow cytometry after staining with FITC-labeled anti NP antibodies. (**B**) Virus infectious activity in the supernatant of infected cells, measured by TCID50 assay. * *p* < 0.05, *** *p* < 0.001, **** *p* < 0.0001—difference between clone (colored asterisk) and original cells is statistically significant, as determined by two-way ANOVA with posterior Sidak test).

To assess the permissiveness of the original and obtained cell lines, cells were infected with a model influenza virus—A/PR/8/34 (H1N1)—and the dynamics of its replication were assessed. Every 24 h, the number of infected cells was evaluated by staining with fluorescently labeled antibodies against virus protein NP, followed by cytometry. The number of newly formed viral particles in the culture medium was determined by titration in highly permissive cells (MDCK). The percentage of infected cells (Figure 9A) was found to be elevated in cell clones F3 and E12. Nonetheless, the titer of newly formed viral particles in the culture medium of the monoclonal cell lines did not differ significantly from the control exceptional for F3, which showed slightly advantage over original WI-38 VA13 cells (Figure 9B).

**Figure 10:**
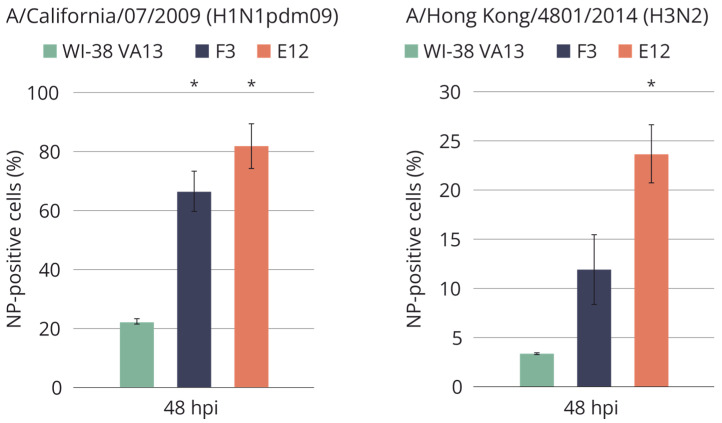
Accumulation of influenza virus nucleoprotein in the original WI-38 VA13 cells (WI-38 VA13) and clones with mutations (F3, F5, E12) after influenza A/California/07/2009 (H1N1pdm09) and influenza A/Hong Kong/4801/2014 (H3N2) virus infection. The amount of NP-positive (infected) cells was measured at specified time points using a flow cytometry method and staining with antibodies against influenza A virus NP. * Difference between clone and original cells is statistically significant at *p* < 0.05 (two-way ANOVA analysis with a posterior Bonferroni criterion).

Cell lines F3 and E12 were also tested for sensitivity to epidemiologically relevant wild-type influenza viruses A/California/07/2009 (H1N1pdm09) and A/Hong Kong/4801/2014 (H3N2). The results indicated an increased percentage of infected cells in the modified cell lines and the percentage was higher as compared to infection with strain A/PR/8/34(H1N1) (Figure 10). Differences in the prevalence of infection between the modified cell lines can be explained by a concomitant impairment of the activation of not only *IFITM3* but also *IFITM1*—this difference was also observed when the cells were infected with influenza virus strain A/PR/8/34(H1N1). In clones with mutations F3 and E12, virus infectious activity in the supernatant of infected cells did not deviate from the original WI-38 VA13 cells.

**Figure 11:**
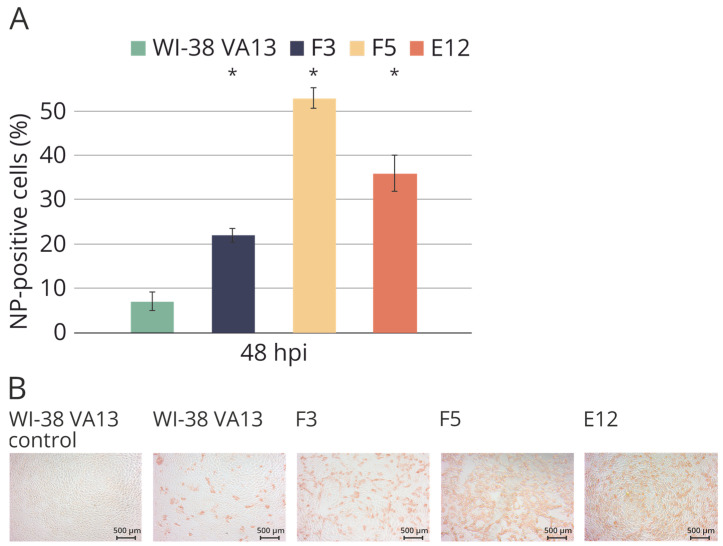
(**A**) Accumulation of influenza virus nucleoprotein in the original WI-38 VA13 cells (WI-38 VA13) and clones with mutations (F3, F5, E12) after influenza A/WSN/33 (H1N1) virus infection. The amount of NP-positive (infected) cells was measured at specified time points using a flow cytometry method and staining with 6D11 monoclonal antibodies against influenza A virus NP. * Difference between clone and original cells is statistically significant at *p* < 0.05 (two-way ANOVA analysis with a posterior Bonferroni criterion). (**B**) Immunostaining of original WI-38 VA13 cells (WI-38 VA13) and clones with mutations (F3, F5, E12) after influenza A/WSN/33 (H1N1) virus infection. The influenza virus NP protein was stained with MAB8258 monoclonal antibody and visualized with HRP-conjugate.

To estimate the permissiveness of the cell lines, we also employed a technique for assessing the accumulation of viral protein NP by means of the number of focus-forming units. Infection with influenza virus A/WSN/33 (H1N1) was carried out at 0.01 TCID_50_ (median tissue culture infectious dose) per cell. It was shown that the knockout of the *IFITM3* gene increases the sensitivity of WI-38 VA13 cells to the influenza A virus; the percentage of infected cells in clones E12 and F5 was higher six- and eight-fold, respectively, relative to wild-type cells (Figure 11).

## 3. Discussion

The study shows that mutations in the *IFITM3* gene lead to elevated sensitivity of human embryonic lung fibroblast subline WI-38 VA13 to infection with an influenza virus. At the same time, differences were observed depending on the virus strain—the cells had the highest sensitivity to infection with strains A/California/07/2009 (H1N1pdm09) and A/WSN/33 (H1N1). Meanwhile, the knockout of *IFITM3* did not significantly affect the number of newly formed viral particles in the culture medium after infection of the cell lines (Figure 9B). It is likely that a knockout of one gene is not enough to effectively suppress the antiviral response system. As a way to overcome this problem, a simultaneous knockout can be performed on several target genes that act at different stages of cell infection with an influenza virus, and this approach will implement comprehensive suppression of innate immune response.

It is also interesting that cell lines F5 and E12, in which the IFITM3 protein is undetectable, differ in the level of sensitivity to the influenza virus. This phenomenon is likely due to the nature of the mutations obtained with the help of the CRISPR/Cas9 system. Transcriptomic analysis of alterations of gene expression revealed that in clone E12, together with downregulation of the mRNA of target gene *IFITM3*, there is downregulation of *IFITM1* mRNA. On the other hand, in clone F5, upregulation of *IFITM2* mRNA was observed but the mRNA levels of genes *IFITM1* and *IFITM3* did not differ significantly from control cells (Figure 5). The combined changes in expression may be caused by dysregulation of transcription of genes in the IFITM family, however, the mechanisms of this transcriptional regulation are currently poorly understood. It is known that there is cis-regulatory enhancer E2-3 located in a region 35 kbp away from the *IFITM3* gene and this enhancer is capable of regulating the expression of the *IFITM1–3* gene group [35]. A mutation at the locus containing *IFITM* genes, such as extended insertions or deletions (as in clone E12), may affect the interaction of the regulatory region with promoters and diminish the mRNA level. As it is known that partial deletion of enhancer E2-3 does not completely repress the genes of the IFITM family, the existence of other enhancers that regulate the transcription of these genes is possible [35]. Nonetheless, their location with respect to genomic locus *IFITM* is currently unknown.

It is also worth noting that in clone E12, significant under-expression of genes of the Jak–STAT pathway was observed, thereby confirming the suppression of innate immune responses in these cells (Figure 8). In clone F5, this effect was not well-pronounced. Differences in the antiviral response between cell lines E12 and F5 are also well illustrated by the heat map based on normalized expression levels of genes in the “response to virus” group (GO:0009615) (Figure 7). The alterations seen in the F5 cell line correlate more with changes in the F3 cell line, in which downregulation of the target protein was demonstrated after mutations in one allele. These findings suggest that reducing the activity of one member of the IFITM family is insufficient for altering the expression of antiviral-response genes. For this alteration to occur, comprehensive repression of several genes is necessary, as observed in the E12 cell line.

In this study, a total of 92 monoclonal cell lines were analyzed into which the genome-editing system was introduced. Among them, only three cell lines with mutations in the *IFITM3* gene were detected and only two cell lines were homozygous for mutant alleles. Furthermore, the proliferation rate of the mutant cell lines was significantly lower than that of the control, and many cell lines even died after several passages. This may mean that this protein affects cell viability and probably participates not only in the regulation of the antiviral response but also (according to the results of our transcriptomic analysis) in the modulation of the processes of cell adhesion, proliferation and cell to cell contacts.

## 4. Materials and Methods

### 4.1. Construction of Plasmids Expressing Components of CRISPR/Cas9 Systems Directed to IFITM3 Gene Knockout

Analysis of gene sequences for the presence of protospacer-associated motifs (PAMs) and the selection of protospacers depending on the parameters of specific and nonspecific activity were performed using the Benchling online tool (available at https://benchling.com/, accessed on 3 February 2021). Protospacers were chosen according to the specificity criterion: the 20-nt sequence was completely complementary only to a region of the *IFITM3* gene; by contrast, during modeling of the interaction of single guide RNA with sequences of other homologous members of the IFITM family (primarily *IFITM2*), only imperfect duplexes formed, with 2–3 mismatches in the seed sequence area. In total, 8 protospacers were selected to construct sgRNAs (Table S1). After that, genetic constructs based on the pSpCas9(BB)-2A-GFP (PX458) vector (Addgene, Watertown, MA, USA, cat. # 48138) were obtained. They encoded single guide RNA with required protospacers (as described in the original article [33]).

### 4.2. Cell Transfection and Cloning

WI-38 VA13 subline 2RA (ATCC #CCL-75.1, derived from the Collection of Vertebrate Cell Cultures, Institute of Cytology, Russian Academy of Sciences, Novosibirsk, Russia) cells were maintained in DMEM/F12 (Thermo Fisher Scientific, Waltham, MA, USA) and supplemented with 10% fetal bovine serum (FBS) (Thermo Fisher Scientific, Waltham, MA, USA) at 37 °C with CO_2_. The cells were seeded into the wells of 6-well plates in 1.5 mL of complete culture medium a day before the transfection. The cell density was 50–80% on the day of transfection. Transfection of cells was carried out using the Lipofectamine^®^ 3000 (Thermo Fisher Scientific, Waltham, MA, USA) reagent in a serum-free growth medium DMEM/F12 (Thermo Fisher Scientific, Waltham, MA, USA). For the transfection, 4 μL of the Lipofectamine reagent was mixed with 1 μg of plasmid constructs based on vector pSpCas9(BB)-2A-GFP (PX458) and the total volume of the mixture was brought to 20 μL with sodium phosphate buffer (pH 7.4). The transfection mixture was incubated for 20 min at room temperature, after which it was added to the cells in the serum-free growth medium. The cells were incubated for 6 h in a CO_2_ incubator at 37 °C. Then, the medium was changed to 1.5 mL of fresh DMEM/F12 supplemented with 10% of FBS. The cells were left in the CO_2_ incubator at 37 °C overnight. The transfection outcome was assessed with the help of the ZOE™ fluorescent cell imaging system (Bio-Rad Laboratories, Hercules, CA, USA). GFP-positive cells were collected 48 h after the transfection on a cell sorter S3e Cell Sorter (Bio-Rad Laboratories, Hercules, CA, USA). The cell suspension was diluted to approximately 100 cells/mL and seeded on 96-well plates (1 cell/well). The clones of individual cells were trypsinized and replicated in 96-well plates for the analysis.

### 4.3. Analysis of the Monoclonal Cell Lines for IFITM3 Gene Mutations

For some monoclonal lines the effectiveness of the genetic modifications introduced by the CRISPR/Cas9 genome-editing system in the *IFITM3* gene was examined by means of endonuclease I of bacteriophage T7. Protospacers three and four were selected so that the site of the expected break introduced by the CRISPR/Cas9 system coincided with the site cleaved by restriction endonucleases ZraI (SibEnzyme, Novosibirsk, Russia) and AatII (SibEnzyme, Novosibirsk, Russia), respectively. The presence of a mutation meant disruption of a restriction site; in this case, DNA is not cut by the restriction endonuclease. This approach enabled us to determine the presence/absence of mutations in the monoclonal cell lines obtained via the transfection with plasmid vectors carrying protospacer three or four. For the restriction analysis, a 20 μL reaction mixture was prepared consisting of 1 μL of an amplicon, 2 μL of restriction endonuclease ZraI for clones with putative mutations in the region of protospacer three (or restriction endonuclease AatII for clones with putative mutations in the region of protospacer four), 2 μL of 10× restriction enzyme buffer and nuclease-free water up to the final volume. The samples were incubated for 1 h at 37 °C. The reaction products were analyzed by electrophoresis in a 1.5% agarose gel.

### 4.4. Detection of Deletions and Insertions in Monoclonal Cell Lines

To determine the presence/absence of deletions and insertions in DNA samples from monoclonal cell lines, the indel detection by amplicon analysis (IDAA) was applied [34]. PCR was conducted with 6-FAM-5′-labeled primers, which implemented one-step fluorophore labeling of amplicons. Next, the amplicons were purified on ExtraGen magnetic particles (GenTerra, Moscow, Russia). PCR products were visualized by electrophoresis in a 1.5% agarose gel. After that, DNA (1–5 ng) was dried on a vacuum evaporator and dissolved in formamide for assessment on genome analyzer ABI Genetic Analyser 3500 (ABI/Life Technologies, Waltham, MA, USA). The data were examined in the GeneMarker (v3.0.1) software (SoftGenetics, State College, PA, USA).

### 4.5. Real-Time Cell Growth Analysis

To evaluate the proliferation rate of the cells carrying mutations in the *IFITM3* gene, a proliferation assay was performed as compared to the original WI-38 VA13 cells with the help of xCELLigence technology on the cell analyzer RTCA xCELLigence DP (ACEA Biosciences, Santa Clara, CA, USA) equipped with E-plate 16 chips (ACEA Biosciences, Santa Clara, CA, USA). The results were analyzed using a RTCA Software (v31.2) software (ACEA Bioscience, Santa Clara, CA, USA). The number of live cells was determined beforehand on a LUNA-II automatic cell counter (Logos Biosystems, Anyang, Republic of Korea) by staining with trypan blue (Bio-Rad Laboratories, Hercules, CA, USA). The cells with viability of at least 90% in three biological replicates obtained by independent defrosting of the cell bank were used for the experiments. Cells were seeded at a density of 20,000/well in the DMEM/F12 medium supplemented with 10% of fetal bovine serum and monitored in real time for 60 h.

### 4.6. Quantification of mRNA and Viral RNA Expression by RT-PCR

Total RNA from WI-38 VA13 cells and modified monoclonal lines were isolated using the Kit for Isolation of Total RNA and MicroRNA from Cells and Tissues (Biolabmix, Novosibirsk, Russia) according to the manufacturer’s protocol. RT-PCR was carried out with the BioMaster RT-PCR SYBR Blue (2×) kit (Biolabmix, Novosibirsk, Russia). The analysis of the results was conducted using a Light Cycler 96 Software (v1.1) software package (Roche, Basel, Switzerland) and qBase+ (v3.4) (Biogazelle, Gent, Belgium). To assess the expression of the *IFITM3* gene, primers IFITM3-F1 5′-CCGTGAAGTCTAGGGACAGG-3′ and IFITM3-R1 5′-CCTGGAAGATCAGCACTGG-3′ were used. As reference signals, *GAPDH* mRNA (GAPDH-F: 5′-GAAGATGGTGATGGGATTTC-3′, GAPDH-R: 5′-GAAGGTGAAGGTCGGAGT-3′) and of 18S rRNA (18S-F: 5′-GATGGTAGTCGCCGTGCC-3′, 18S-R: 5′-GCCTGCTGCCTTCCTTGG-3′) were quantitated. To estimate the level of viral mRNA, the primers allowing to detect fragment of the *M* gene of the influenza A were used: FluA-F 5′-CCCTCAAAGCCGAGATCGC-3′ and FluA-R 5′-AGGTGACARRATTGGTCTTGTCTTTA-3′. The primers for *IFITM3* mRNA and Influenza A RNA detection contained phosphoryl guanidine groups.

### 4.7. Detection of the IFITM3 Protein in the Monoclonal Cell Lines by Western Blotting

Proteins were isolated from a pool of cells (2.5 × 10^5^) via the addition of 50 μL of RIPA lysis buffer to a cell pellet, incubated for 30 min on ice, then centrifugation for 20 min at 4 °C and 13,000 relative centrifugal field units and then the pellet was discarded. The cell lysates were stored at −20 °C. Proteins were separated by protein electrophoresis in a 10% polyacrylamide gel; 10 μL of Spectra™ Multicolor Broad Range Protein Ladder (Thermo Scientific, Waltham, MA, USA) served as molecular-weight markers. After that, the proteins were electrotransferred onto a nitrocellulose membrane and Western blotting was performed in an IBind system (Thermo Scientific, Waltham, MA, USA). Primary antibodies were rabbit anti human IFITM3 antibody (1:200) (VPA00312, Bio-Rad Laboratories, Hercules, CA, USA) and rabbit anti GAPDH antibody (1:1000) (ab181602, Abcam, Cambridge, UK). The secondary antibody was rabbit IgG horseradish peroxidase-conjugated antibody (1:200) (HAF008, R&D Systems, Minneapolis, MN, USA). To visualize the results, the membrane was incubated in a luminol solution. Next, the membrane was washed with deionized water. Imaging was implemented on an Amersham Imager 600 device (GE Healthcare Life Sciences, Chicago, IL, USA).

### 4.8. Infection of Cells with the Influenza A Virus

Several influenza viruses were used in the study. Well known influenza virus strain A/PR/8/34 (H1N1) was obtained from the Collection of Influenza and Acute-Respiratory-Disease Viruses at the Smorodintsev Research Institute of Influenza (St. Petersburg, Russia). Another laboratory influenza strain A/WSN/30 (H1N1) was kindly provided by Professor N.V. Kaverin (Ivanovsky Institute of Virology, Moscow, Russia). Wild-type influenza viruses A/California/07/2009 (H1N1pdm09) and A/Hong Kong/4801/2014 (H3N2), were obtained from CDC (Atlanta, GA, USA). All viruses were cultured in 10–12-day-old chicken embryos. Infectious activity of viruses was determined by titration in MDCK cells (obtained from the International Reagent resource, #FR-58), as follows: Monolayer of MDCK cells in a 96-well plate were infected with serial 10-fold dilutions of a virus (in rows, 4 wells per dilution) in the serum-free medium supplemented with 2.5 μg/mL TPCK trypsin (Sigma-Aldrich, Burlington, MA, USA) and incubated at 37 °C and 5% of CO_2_ for three days. Virus replication in particular wells was assessed by hemagglutination reaction of culture medium with a 0.5% suspension of chicken erythrocytes. The infectious activity was calculated by the Reed–Muench method and expressed as TCID_50_/mL.

Influenza virus experiments were performed using original WI-38 VA-13 cells (obtained from the Collection of Vertebrate Cell Cultures, Institute of Cytology, Russian Academy of Sciences, Novosibirsk, Russia) as well as with the CRISPR-Cas9 modified cell clones obtained within this study. Virus growth rate at different time points was evaluated by three parameters: newly produced virus particles after cell infection (infectious activity), number (proportion) of infected cells measured by FACS and number (proportion) of infected cells measured by focus assay. Comparisons were made of virus growth in modified cells versus original strain at each time point. ANOVA with posterior pairwise comparisons was used to determine statistical significance of the observed differences. The level of significance was set at 0.05. Virus growth curve experiments were performed using dense monolayer cells grown in 12-well plates. Prior to infection, the cells were washed with the serum-free medium supplemented with 1 μg/mL TPCK trypsin. A virus dilution was prepared in the aforementioned medium so that multiplicity of infection was 0.01 TCID_50_/cell, considering a monolayer density of 1.3 × 10^5^ cells/cm^2^ and an inoculum volume of 250 μL/well. The inoculum was added to cells, incubated for 1 h at 37 °C, 5% of CO_2_, and then removed. After that, 2 mL/well of serum-free media with trypsin was added and plates were incubated at 37 °C and 5% of CO_2_. The experiment was setup with three wells as three biological repeats. At 24, 48 and 72 h after the infection, culture supernatant was collected and titrated in MDCK cells to analyze the infectious activity of newly generated viruses. Infected cells were also collected by trypsin-EDTA (Sigma-Aldrich, Burlington, MA, USA), fixed, permeabilized (BD Cytofix/Cytoperm) and stained with fluorescently labeled antibodies to the NP protein of influenza A virus (clone 6D11-FITC, PPDP Ltd., St Petersburg, Russia; dilution 1:1000) to analyze the amount of productively infected cells. Uninfected cells served as a control in the staining procedure. The percentage of infected cells was determined on a Cytoflex flow cytometer (Beckman Coulter, Brea, CA, USA).

To assess the permissiveness of cells to infection by focus assay, based on accumulation of viral NP protein (nucleoprotein), cells were grown in a 96-well plate in quadruplicate to a density of 50 × 10^3^ cells/well. Before infection, the cells were placed in the serum-free growth medium supplemented with 1 μg/mL trypsin. The virus dilution was prepared so that the infection dose was 0.01 TCID_50_/cell. After addition of the virus, the cells were incubated for 24 h at 37 °C, 5% of CO_2_. Next, the cells were fixed with acetone, after which 50 μL (1:2500 dilution) of a primary antibody against the NP protein of the influenza A virus [mouse anti-influenza A monoclonal antibody, MAB8258 (Millipore, Burlington, MA, USA)] was added to the cells and incubated at 37 °C for 1 h. After that, cells were washed twice with PBS, and 50 μL (1/4000 dilution) of a biotinylated secondary antibody was added into the wells [Anti-Mouse IgG (Fab specific)–Biotin antibody, B7151 (Sigma-Aldrich, Burlington, MA, USA)] and incubated at 37 °C for 30 min. The cells were washed twice with PBS, and 50 μL (1:4000 dilution) of streptavidin conjugated with horseradish peroxidase (Sigma-Aldrich, Burlington, MA, USA) was added and incubated at 37 °C for 30 min. The cells were washed twice with PBS, and 50 μL of AEC buffer was introduced. The multi-well plates were left in a dark place at room temperature for 10–15 min until coloring appeared. The number of focus-forming units (number of stained cells) was estimated in the ImageJ (v1.53) software.

### 4.9. Analysis of Transcriptome-Sequencing Data

Construction of cDNA libraries was conducted according to a standard protocol based on the NEBNext Ultra II Directional library preparation kit (New England Biolabs, Ipswich, MA, USA) and a module for enrichment of the poly(A) fraction of RNA (New England Biolabs, Ipswich, MA, USA), followed by mass parallel sequencing on the NextSeq Illumina platform at the Institute of Fundamental Medicine and Biology at Kazan Federal University (Kazan, Russia). Paired-end reads of 75 nucleotides each were obtained by means of the NextSeq 500/550 High Output v2.0 Kit (150 cycles) (Illumina, San Diego, CA, USA).

The raw data were saved as FASTQ format files. The quality control of the raw and trimmed reads was performed using FastQC (v0.11.9) and MultiQC (v1.9) [36,37]. Trimming of the adapter content and quality trimming was performed using fastp (v0.21.0) [38]. The reads complementary to ribosomal RNA were filtered out from the trimmed reads using SortMeRNA (v2.1b) [39]. The filtered reads were aligned via STAR (v2.7.7a) to a combined genomic reference of the human genome (GRCh38) and the genome of influenza A/Puerto Rico/8/1934 (H1N1) [40]. Mapped reads were visualized using the Integrative Genomics Viewer (IGV) (v2.8.0) [41]. The counting reads were performed with the featureCounts function from the R package Rsubread (v2.4.3) [42]. Differentially expressed RNAs were identified using R package DESeq2 (v1.30.1) with a FDR-adjusted *p*-value < 0.05 and the absolute value of a log2(FC) > 0.58 [43]. RNA-seq data have been deposited in the ArrayExpress database at EMBL-EBI (https://www.ebi.ac.uk/biostudies/arrayexpress, accessed on 10 July 2023) under accession number E-MTAB-9511 (virus infected original WI-38 VA-13) and E-MTAB-13582 (virus infected original WI-38 VA-13 and clones with mutations in the *IFITM3* gene (F3, F5, E12)) [44].

## Figures and Tables

**Table 1:** The number of genes differentially expressed in original WI-38 VA13 cells and clones with mutations (F3, F5, E12) at 24 h after influenza A virus infection (relative to uninfected cells) (FDR-adjusted *p*-value < 0.05, absolute value of a LogFC > 0.58).

Sample	Regulation	Number
WI-38 VA13 (24 hpi vs. 0 hpi)	Upregulated	177
	Downregulated	3
F3 (24 hpi vs. 0 hpi)	Upregulated	364
	Downregulated	54
F5 (24 hpi vs. 0 hpi)	Upregulated	397
	Downregulated	43
E12 (24 hpi vs. 0 hpi)	Upregulated	965
	Downregulated	334

## Data Availability

RNA-seq data have been deposited in the ArrayExpress database at EMBL-EBI (https://www.ebi.ac.uk/biostudies/arrayexpress, accessed on 10 July 2023) under accession number E-MTAB-9511 (virus infected original WI-38 VA-13) and E-MTAB-13582 (virus infected original WI-38 VA-13 and clones with mutations in the *IFITM3* gene (F3, F5, E12)).

## Supplementary Materials

The following supporting information can be downloaded at: https://www.mdpi.com/article/10.3390/ijms25010625/s1.
