# Supplementary material for: A Knockout of the IFITM3 Gene Increases the Sensitivity of WI-38 VA13 Cells to the Influenza A Virus
Source: Int J Mol Sci. 2024 Jan 3;25(1):625. doi: 10.3390/ijms25010625 (PMC10778886; doi:10.3390/ijms25010625)
Supplement: Supplementary file 1 [file ijms-25-00625-s001.zip › ijms-2788171-supplementary.pdf]

Table S1. Protospacer sequences.

Guide	Target Sequence	PAM
gRNA1	TGAATCACACTGTCCAAACC	TGG
gRNA2	GTCAACAGTGGCCAGCCCCC	AGG
gRNA3	ACGTCAGTGGCTTTGTCTGT	GGG
gRNA4	TGTGGATCACGGTGGACGTC	GGG
gRNA5	TGGATCACGGTGGACGTCGG	GGG
gRNA6-1	GTGCTGATCTTCCAGGCCTA	TGG
gRNA6-2	TAGGCCTGGAAGATCAGCAC	TGG
gRNA6-3	AGGCCTGGAAGATCAGCACT	GGG
